# Testicular Epidermoid Cyst: A Rare Case Report

**DOI:** 10.1016/j.ijscr.2022.107167

**Published:** 2022-05-04

**Authors:** Reza Maulana, Safendra Siregar, Bethy S. Hernowo

**Affiliations:** a Urology Department, Hasan Sadikin Academic Medical Center, Universitas Padjadjaran, Bandung, Indonesia; b Urology Department, Universitas Syiah Kuala, Aceh, Indonesia; c Pathology Anatomy Department, Hasan Sadikin Academic Medical Center, Universitas Padjadjaran, Bandung, Indonesia

**Keywords:** Case report, Cyst, Epidermoid, Testicular mass

## Abstract

**Introduction an importance:**

Testicular epidermoid cysts (TECs) are rare benign testicular neoplasms. Recently, testicular epidermoid cysts (TECs) are listed as teratoma of prepubertal type, however it is still difficult to differentiate the epidermoid cyst from malign testicular tumor. Therefore, we would like to report testicular epidermoid cyst at our institution.

**Case presentation:**

A 67-year-old man from Indonesia, presented with chronical painless mass of testis since one year ago. On physical examination obtained normal penile structure with descended testicles, palpable intrascrotal mass with size of 10 × 7 × 5 cm, firm consistency, immobile, without any tenderness, and no lymphadenophaty in groin. Scrotal USG showed intratesticular mass, homogenous parenchym, showed no vascularization during Doppler examination. Histopathological examination revealed the specimen of right scrotum with size of 12.5 cm × 8.5 cm × 6.1 cm with red-brownish colored, during lamellation, obtained encapsulated mass with size of 12.2 cm × 7.9 cm × 6 cm, hollowed space filled with porridge-like texture with capsule thickness of 0.1–0.3 cm.

**Clinical discussion:**

Epidermoid cysts are benign lesions occurring on the skin usually, however, it rarely occurs in intratesticular area. Most of the cases (60%) presented with the typical onion-ring phenomenon. Histopathological findings commonly revealed typical well-defined cyst lined by a fibrous membrane. No skin appendages are found in the cyst's lumen and no germ cell neoplasm (GCN) is present in the adjacent testicular parenchyma.

**Conclusion:**

All testicular masses are considered malignant until proven otherwise. It is necessary to do accurate diagnosis for the prevention of unnecessary radical orchiectomy.

## Highlights

• All testicular masses are considered malignant until proven otherwise.
• It is necessary to do accurate diagnosis for the prevention of unnecessary radical orchiectomy.
• Histopathological findings of testicular epidermoid cyst commonly revealed typical well-defined cyst lined by a fibrous membrane and filled with layers of cornifying squamous epithelium and cell debris.

## 1 Introduction and importance

Epidermoid cysts are benign masses of epithelial hyperplasia which are commonly found in hair-bearing areas [1]. However, testicular epidermoid cysts are still a rare cause of testicular pathology which only responsible for 2.1% of all testicular masses [2].

Epidermoid cysts are typically discrete spherical or oval lesions. Histologically, epidermoid cysts are true cysts with keratin layers encapsulated with a fibrous capsule and lined with simple squamous epithelium. Calcification may also be seen within the wall or the cyst [3].

The variable appearance combined with the rarity of presentation makes it challenging to establish a diagnosis of an epidermoid cyst from clinical examination and sonographic findings alone. It is essential to differentiate these benign cysts from solid intratesticular masses as the latter are invariably malignant. Although benign testicular lesions can be enucleated or managed conservatively, malignant lesions often require orchiectomy [4]. Therefore, we would like to report testicular epidermoid cyst at our institution.

This case report has been reported in line with the Surgical Case Report (SCARE) guidelines [5].

## 2 Case presentation

A 67-year-old man from eastern Purwakarta, Jawa Barat, Indonesia, presented with chronical painless mass of testis since one year ago. Currently, the mass was getting larger in size and become firm in consistency. This complaint was accompanied by significant weight loss within two months. Patient also complained for frequent urination with weak stream and dribbling since 3 years ago and the complaint become worst during this 5 months. There was no history of fatigue, malaise, dyspnea, loss of consciousness, trauma, fever, infertility, lymphadenitis, lymphangitis, or previous history of testicular tumor. There was no history of hematuria and stone within the urine. There was no history of allergies. Patient had history of urinary catheter insertion since 5 days before when he was admitted to inpatient care of hospital nearby his hometown. Since the catheter was being removed, patient complained of anuria. Patient had history of controlled Diabetes Mellitus since one year ago. No previous history of hypertension. There was no history of similar disease in family.

**Fig. 1:**
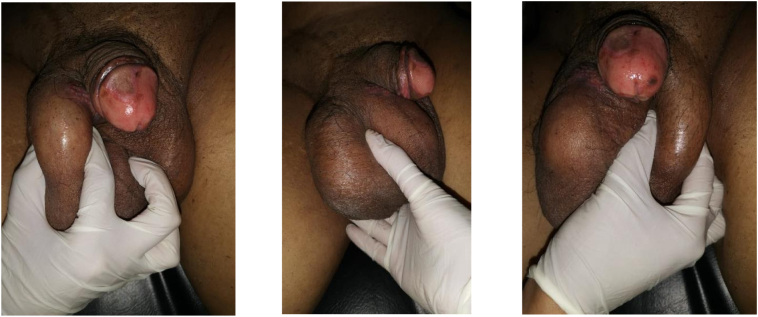
Macroscopic appearance of intrascrotal mass.

On physical examination obtained normal penile structure with descended testicles, palpable intrascrotal mass with size of 10 × 7 × 5 cm, firm consistency, immobile, without any tenderness, and no lymphadenophaty in groin (Fig. 1). Palpation of the bilateral testis and spermatic cords were found no remarkable abnormality. There was no inguinal lymphadenopathy.

The laboratorium tests revealed anemia with Hb was 10.8 g/dL and Hematocrite was 29.2%, white blood cells of 8600 cells/μL, Platelet was 246,000 cells/μL. Ureum and creatinine were increasing, with ureum was 77 mg/dL and Creatinine was 2.60 mg/dL. Natrium was 137 mEq/L and Kalium was 3.5 mEq/L. Random blood glucose within normal limit (109 mg/dL).

**Fig. 2:**
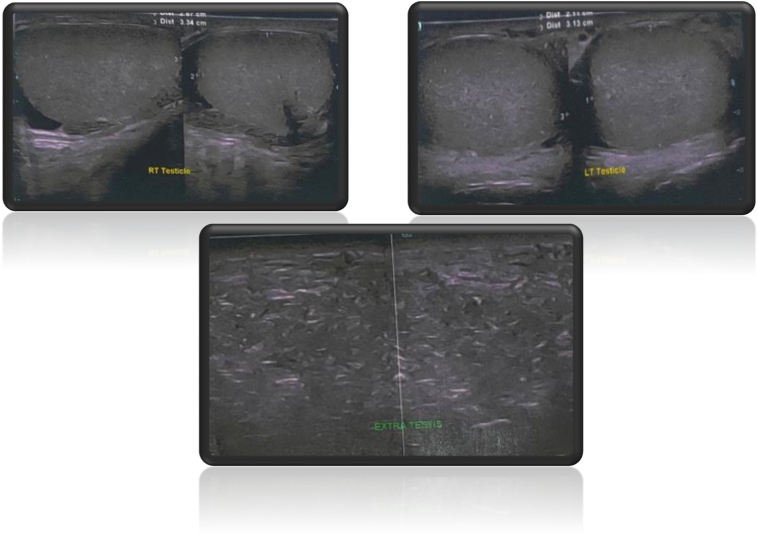
Ultrasonography of scrotum and intrascrotal mass.

Scrotal USG showed intratesticular mass, without peristaltic, homogenous parenchym, showed no intralesion vascularization during Doppler examination. The right and left testis, bilateral epididymis, and bilateral spermatic cords were of normal size, shape, and echotexture (Fig. 2).

**Fig. 3:**
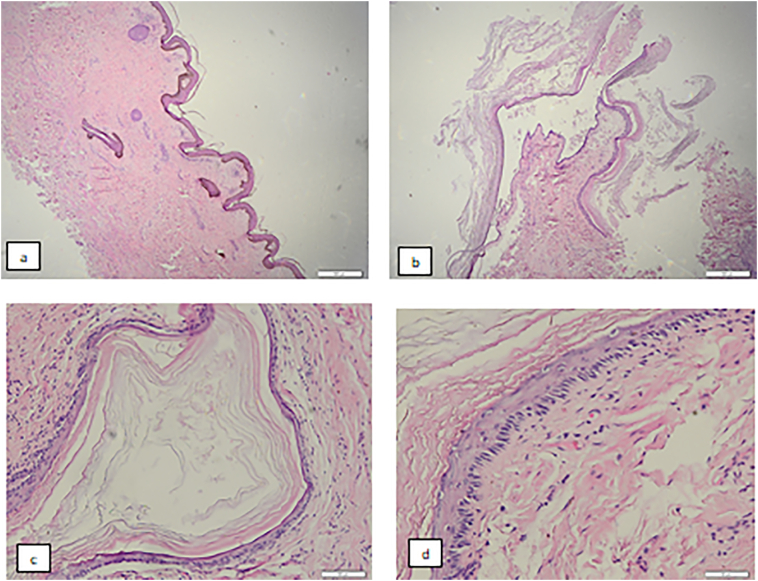
The scrotum dextra preparation is lined with stratified squamous epithelium with keratinized skin with the dermis consisting of fibrocollagenous connective tissue, including skin adnexa within normal limits (a). Visible cyst structure with cyst wall lined with stratified squamous epithelium with cell nuclei within normal limits. Inside the lumen contains a mass of keratin (b, c, d).

After assessing the testicular mass, an informed consent was taken. Thereafter left trans-scrotal orchiectomy was performed by urologist resident who experience in this field for around 3 years in operating room and specimen sent to pathology anatomy department. Histopathological examination revealed the specimen of right scrotum with size of 12.5 cm × 8.5 cm × 6.1 cm with red-brownish colored, during lamellation, obtained encapsulated mass with size of 12.2 cm × 7.9 cm × 6 cm, hollowed space filled with porridge-like texture with capsule thickness of 0.1–0.3 cm. Microscopically, right scrotum was covered with keratinized simple squamous epithelium, nuclei within normal limit. Subepithelial revealed fibrocollagenous connective tissue with inflammatory cells, lymphocyte, and histiocyte. Adnexa and hair follicle within normal limit. Cysts were found covered with simple squamous cell (Fig. 3). After a week after patient discharged, patient came to outward clinic and no complication was found. Patient feels better in carrying out daily activities.

## 3 Clinical discussion

Epidermoid cysts are benign lesions occurring on the skin usually, however, it rarely occurs in intratesticular area [6]. Epidermoid cysts are rare but it become the second most common benign tumors of the testes, mostly found within the second to fourth decades of life [7]. The most common symptom is the present of a palpable painless testicular mass, which raises the clinical suspicion of malignancy [7]. According to Anheuser et al., testicular epidermoid cysts (TECs) have many clinical characteristics in common with GCT: both of them mostly presented with painless mass, the age predisposition of early adulthood and the predominance of the right side [8].

The differentiation of a benign epidermoid cyst from a malignant tumor is important to prevent an unnecessary orchiectomy. This differentiation could be made by assessing with B-mode and color Doppler sonography, this assessment become essential, but often not confidently able to establish the correct diagnosis. Further biochemical and imaging investigations have been described to improve diagnostic confidence [7].

Tumor marker test results (α-fetoprotein and β-human chorionic gonadotropin) are usually negative for an epidermoid cyst, but a primary germ cell tumor of the testis also may not have elevated tumor marker values. Magnetic resonance imaging characteristics of epidermoid cysts normally mirror the B-mode sonographic appearances [9]. There is no clear advantage of unenhanced magnetic resonance imaging in the characterization of epidermoid tumors; however, contrast enhanced magnetic resonance imaging shows a lack of enhancement, a feature adequately depicted by contrast enhanced sonography with advantages related to accessibility, cost, and repeatability [9].

Most of the cases (60%) presented with the typical onion-ring phenomenon [10], [11]. The cyst-like appearance and the roundish configuration are further typical findings [8]. A more distinctive finding is the absence of vascularization of the cyst core documented with CCDS (color-coded duplex sonography) [12]. Scrotal MRI can likewise detect these morphologic features [13]. The avascular nature of the cyst core is shown by the absence of signal enhancement after application of contrast [13].

Local excision (TSS) is the treatment of choice for TEC. Orchiectomy is exceptionally required in cases with a large mass and only little remaining testicular tissue [8]. Histopathological findings commonly revealed typical well-defined cyst lined by a fibrous membrane and filled with layers of cornifying squamous epithelium and cell debris. No skin appendages are found in the cyst's lumen and no germ cell neoplasm (GCN) is present in the adjacent testicular parenchyma [8]. If the pathology is reported as benign the procedure can be terminated, if the final pathology describes a malignant pathology, radical orchiectomy is required [13].

## 4 Conclusion

All testicular masses are considered malignant until proven otherwise. It is necessary to do accurate diagnosis for the prevention of unnecessary radical orchiectomy.

## Sources of funding

No source of funding.

## Ethical approval

Ethical approval for this study was obtained from Padjadjaran University.

## Consent

Written informed consent was obtained from the patient for publication of this case report and accompanying images. A copy of the written consent is available for review by the Editor-in-Chief of this journal on request.

## Author contribution

Kiagus Ferry: Analysis and interpretation of data, drafting the article.

Ferry Safriardi: Analysis and interpretation of data, drafting the article.

Sawkar Vijay Pramod: Analysis and interpretation of data, drafting the article.

Bethy S Hermowo: Analysis and interpretation of data, drafting the article,

All authors have read and approved the manuscript.

## Research registration

Not applicable.

## Guarantor

Reza Maulana.

## Provenance and peer review

Not commissioned, externally peer-reviewed.

## Declaration of competing interest

None.
